# Development and initial validation of a Swedish inventory to screen for symptoms of deficient perineum in women after vaginal childbirth: ‘Karolinska Symptoms After Perineal Tear Inventory’

**DOI:** 10.1186/s12884-022-04964-w

**Published:** 2022-08-13

**Authors:** Emilia Rotstein, Philip von Rosen, Sofie Karlström, Jona Elings Knutsson, Nina Rose, Ellinore Forslin, Per J. Palmgren, Gunilla Tegerstedt, Hedvig Engberg

**Affiliations:** 1 Department of Clinical Science Intervention and Technology, Karolinska Institutet, Stockholm, Sweden; 2 Department of Gynecology and Reproductive Medicine, Karolinska University Hospital, Stockholm, Sweden; 3 Department of Neurobiology, Care Sciences and Society, Division of Physiotherapy, Karolinska Institutet, Stockholm, Sweden; 4 Department of Women’s and Children’s Health, Karolinska Institutet, Stockholm, Sweden; 5 Department of Obstetrics and Gynecology, Hospital of Västmanland Västerås, Västerås, Sweden; 6 Department of Learning, Informatics, Management and Ethics, Karolinska Institutet, Stockholm, Sweden

**Keywords:** Design, Questionnaire, Perineum, care, Postpartum, Pelvic floor, Disorder, Assessment, Patient outcome, Psychometrics, Sexual dysfunction, Physiological, Pelvic floor

## Abstract

**Background:**

Perineal tears are common after vaginal birth and may result in pelvic floor symptoms. However, there is no validated questionnaire that addresses long-term symptoms in women with a deficient perineum after vaginal birth. Thus, the objective of this study was to develop and psychometrically evaluate a clinical screening inventory that estimates subjective symptoms in women with a deficient perineum more than one year after vaginal delivery.

**Material and methods:**

The development and psychometric evaluation employed both qualitative and quantitative methods. Qualitative strategies involved content validity and Think Aloud protocol for generation of items. The psychometric evaluation employed principal component analysis to reduce the number of items. The inventory was completed by women with persistent symptoms after perineal tears (*N* = 170). Results were compared to those of primiparous women giving birth by caesarean section (*N* = 54) and nulliparous women (*N* = 338).

**Results:**

A preliminary 41-item inventory was developed, and the psychometric evaluation resulted in a final 11-item inventory. Women with confirmed deficient perineum after perineal trauma scored significantly higher on the symptoms inventory than women in control groups. A cut-off value of ≥ 8 could distinguish patients from controls with high sensitivity (100%) and specificity (87–91%).

**Conclusions:**

The Karolinska Symptoms After Perineal Tear Inventory, is a psychometrically valid 11-item patient-reported outcome measure for symptoms of deficient perineum more than one year after vaginal birth. More research is needed to validate the inventory in various patient populations as well as its use in pelvic floor interventions. The inventory has the potential to improve patient counseling and care in the future.

**Supplementary Information:**

The online version contains supplementary material available at 10.1186/s12884-022-04964-w.

## Background

It is well known that women suffer from perineal morbidity and pelvic floor dysfunction (PFD) e.g., urinary or fecal incontinence, sexual dysfunction, pelvic pain, and/or pelvic organ prolapse after vaginal childbirth [1]. Moreover, PFD negatively affects women’s quality of life including their social, emotional, and sexual well-being [2]. It has previously been assumed that women with severe perineal tears have the highest risk of morbidity compared to women with a first- or second-degree perineal tear, thus, research has focused on long-term consequences for women after third- or fourth-degree tears [3]. Regardless of severity of the perineal tear, studies indicate that women can experience symptoms of PFD, such as a feeling of wide vagina, bowel emptying difficulties, dyspareunia, and perineal pain many years after vaginal birth [4–6]. This may possibly be due to a deficient perineum causing a loss of support of the pelvic floor.

Even though the prevalence of second-degree perineal tears is high among both primiparous (35–78%) and multiparous women (35–40%) [7], there is a scarcity of research on postnatal recovery in general [8, 9], and in particular on symptoms more than one year after vaginal birth. Moreover, a recent review highlights the lack of instruments designed for evaluating women with PFD symptoms postpartum [10].

The lack of research affects the level of clinical follow-up offered to women with non-obstetric anal sphincter injury (non-OASI) [6]. In Sweden, most women with a third- or fourth-degree tear (OASI) are offered registry-based follow-up eight weeks and one-year post-partum through the Swedish National Quality Registry of Obstetric Tears, nevertheless, validated measures for symptoms of a deficient perineum are lacking. In Stockholm all women with OASI are offered followed-up by an obstetrician and/or physiotherapist, whereas women with non-OASI are offered a general postpartum visit with a midwife 8–12 weeks postpartum. Results from a qualitative study emphasize that women lack information about what symptoms should receive medical attention after vaginal childbirth [11], which is also in line with our clinical experience from the Karolinska Pelvic Floor Center. Furthermore, a recent report by The Swedish Agency for Health Technology Assessment and Assessment of Social Services on women’s experiences after perineal trauma, highlights that women want direct and specific questions regarding symptoms of PFD after childbirth [12]. Similarly, urogynecology providers fail to educate patients about protective factors and risk factors for PFD after childbirth and believe that an easy-use screening questionnaire would be helpful in clinical practice [13]. According to the International Consultation on Incontinence, the most valid measures of presence, severity, and impact of PFD are psychometrically validated and self-completed patient inventories [14].

The overall aim of this study was to construct an accessible and valid instrument to improve quality of care for women after vaginal birth. The specific objective was to describe the development of a novel inventory and psychometrically evaluate it to measure the presence of symptoms in women with deficient perineum more than one year after vaginal birth.

## Material and methods

Development and psychometric evaluation of the new inventory was conducted employing both qualitative and quantitative strategies. The inventory development was conducted in four steps according to Streiner et al. [15]. For the psychometric evaluation principal component analysis (PCA) was employed to reduce the number of items in the inventory, while preserving as much variation of data as possible [16]. We then compared three different groups to assess construct validity [17]. First, we hypothesized that the items would capture symptoms in women with confirmed deficient perineum assessed at the Karolinska Pelvic Floor Center by pelvic exam and three-dimensional ultrasonography, hereafter called Patient group. Second, we hypothesized that the Patient group would score significantly higher on a symptoms inventory compared to primiparas who have given birth by elective caesarean section, and to non-pregnant nulliparous women. The study was performed in line with the principles of the Declaration of Helsinki. All participants gave their oral and written informed consent and were informed that they could withdraw from the study at any time. Approval was granted by the Regional Ethics Committee of Stockholm, Sweden on 24 May 2013 (reference number: 2013/445–31/4).

### Development of the inventory

#### Step 1: Literature review

To conceptualize and define the field, a review of the literature was performed with the aim of identifying existing validated instruments for symptoms of a deficient perineum after obstetric perineal trauma. The scientific literature was searched in PubMed and Web of Science and included all articles published until 2017. An additional literature search was performed in December 2021 for any new articles published. Original publications, including empirical and review articles written in English and Swedish, were included, whereas conference papers, theses and presentations were excluded. However, no publication had constructed or validated an inventory evaluating persistent symptoms of a deficient perineum after obstetric perineal trauma more than one year after vaginal birth.

#### Step 2: Generation of items

Seven symptom domains were identified based on findings from a previous qualitative study exploring symptoms in women diagnosed with a deficient perineum after second-degree perineal tears [18]. Furthermore, to see if other symptoms domains should be added, existing inventories for perineal trauma and related conditions (e.g., OASI, sexual dysfunction, and pelvic organ prolapse) were examined but no new domain was found. In conclusion, the item generation in step 1 and step 2 resulted in 17 items and encompassed questions or statements concerning: a feeling of wide vagina, vaginal flatulence, bowel symptoms, a bearing down sensation, pain, sexual dysfunction, and quality of life. A 4-point Likert response format (0–3) was used for answers to each item: 0 = *Strongly disagree, 1* = *Disagree, 2* = *Agree,* and *3* = *Strongly agree* when the item involved a constant condition, and 0 = *Never, 1* = *Sometimes, 2* = *Often,* and *3* = *Always* when the item related to a fluctuating condition.

#### Step 3: Content validity, advisory panel

The recommended number of experts to review an instrument varies, but at least five assessors have been suggested to sufficiently review an instrument with adequate control over chance agreement [19]. Therefore, an advisory panel was recruited consisting of five Swedish urogynecologists (not employed by the Karolinska University Hospital, nor members of the research team) with vast clinical and scientific experience in PFD. The advisory board graded the questions in the items pool according to Lawshe et al. and suggested new questions of clinical importance [20]. Each item was thus scored by the assessors using a 4-point Likert response: *1* = *not relevant, 2* = *has some relevance*, *3* = *somewhat relevant but needs revision,* and *4* = *highly relevant*. A content validity ratio (CVR) was calculated for each question. The formula for the CVR is CVR = (Ne – N/2)/(N/2), where Ne = number of experts indicating an item as “4” and *N* = the total number of experts. CVR varies between 1 and − 1, and a higher score indicates greater agreement among panel members. Questions with a CVR > 0 were considered to have content validity [19]. Following the review of the advisory panel two items exhibited CVR scores < 0 and were dismissed. The remaining 15 items displayed CVR values ranging between 0.2–1, thus indicating agreement among panel members. The advisory panel added an additional five questions regarding anal incontinence and sexual dysfunction resulting in a total of 20 items.

#### Step 4: Cognitive interviews

When creating the inventory, we paid careful attention to coherence between questions and answering alternatives. As part of the validation process, cognitive interviews using the “Think Aloud” technique were carried out in order to partly ensure the instrument’s face validity [21]. The Think Aloud method encourages respondents to verbalize what their thoughts are while answering each item. The interviewer records what is said and simply supports the respondent as she thinks aloud. Thus, the method was used to determine: 1) if respondents understood the item; 2) if respondents understood the item the way the researcher intended, and 3) how respondents calibrated the item and its response options [22].Three Swedish-speaking women, aged 30–38, with completed non-medical university degrees, and with subjective symptoms after second-degree perineal tears were recruited. Separate interviews were performed with each woman. They read one item at a time and were asked to think aloud and with their own words describe the question or statement. When all items had been covered, a Think Aloud and probing methodology was employed where the participants provided feedback on the entire inventory. The interviews were audio-recorded and transcribed verbatim. The questions were then rephrased or divided accordingly into multiple questions, and response options were changed. In total 21 new items were added. The accumulated feedback on item relevance, content, and wording, resulting in removal, replacement, and rewording of the items, produced a preliminary version of the inventory that included seven symptom domains with a total of 41 items (Table 1): A feeling of wide vagina (5 items), vaginal flatulence (8 items), bowel symptoms (8 items), bearing down sensation (4 items), pain (3 items), sexual dysfunction (12 items), and quality of life (1 item).

**Table 1:** Development of the preliminary 41 items and steps taken for item reduction

Development of the inventory					Psychometric evaluation			Final version
**Constructed items**	**CVR**	**Advisory Panel**	**Think aloud**	**Item**	**PCA** **Components**	**Clinical** **round table**	**PCA** **Correlations**	
Do you feel that your vaginal opening is too wide/loose or too big?	1	No changes	*Do you feel that your vagina is too wide/loose?	1	Component 7	Retained	*r* < 0.6 for all items	Retained
Do you have a feeling of looseness deeper down, inside the vagina?	0.6	No changes	*Do you have a feeling of looseness deep inside the vagina?	2	Component 7	Retained	*r* < 0.6 for all items	Retained
Do you feel that your vaginal opening is too narrow/tight or too small?	-0.2	Dismissed due to CVR < 0						
Are you experiencing discomfort because you feel your vaginal opening is exposed or unprotected?	0.6	No changes	No changes	3	Component 7	Dismissed		
			+ Are you experiencing that your genital area is cold because your vaginal opening is exposed or unprotected?	4	Component 10	Dismissed		
			+ Are you bothered by air or a sensation of draft in the genital area?	5	Component 1	Dismissed		
Are you bothered by air filling the vagina?	0.6	No changes	*Are you bothered by air entering the vagina?	6	Component 1	Retained	Correlated (*r* = 0.62) with item 9	Retained
			+ Are you bothered by air entering the vagina when you are at rest?	7	Component 1	Dismissed		
			+ Are you bothered by air entering the vagina during exertion?	8	Component 1	Dismissed		
			+ Are you bothered by air entering the vagina when you have sex?	9	Component 8	Retained	Correlated (*r* = 0.62) with item 2	Dismissed
Are you bothered by sounds caused by air escaping from the vagina (vaginal flatulence)?	1	No changes	No changes	10	Component 1	Retained	*r* < 0.6 for all items	Retained
			+ Are you bothered by vaginal flatulence while at rest?	11	Component 1			
			+ Are you bothered by vaginal flatulence during exertion?	12	Component 1	Dismissed		
			+ Are you bothered by vaginal flatulence during sex?	13	Component 8	Dismissed		
			+ Do you experience difficulties defecating?	14	Component 4	Dismissed		
Do you need to use your fingers to apply pressure from inside the vagina or around the anus to empty your bowel?	1	No changes	*Do you need to use your fingers to apply pressure from inside the vagina or around the anus to defecate?	15	Component 9	Retained	*r* < 0.6 for all items	Retained
			+ Do you need to use your fingers to apply pressure from inside the vagina or around the anus to release anal flatulence?	16	Component 4			
Do you have to sit or stand in a particular position to be able to defecate?	0.6	No changes	No changes	17	Component 4	Retained	*r* < 0.6 for all items	Retained
			+ Does it make it easier to defecate if you sit or stand in a particular position?	18	Component 4	Dismissed		
		+ Are you bothered by flatus incontinence?	No changes	19	Component 6	Dismissed		
		+ Are you bothered by leakage of loose stool?	No changes	20	Component 6	Retained	*r* < 0.6 for all items	Retained
		+ Are you bothered by leakage of solid stool?	No changes	21	Component 6	Dismissed		
Are you bothered by a feeling of heaviness in the genital area?	1	No changes	No changes	22	Component 3	Retained	*r* < 0.6 for all items	Retained
			+ Are you bothered by a feeling of heaviness in the genital area while at rest?	23	Component 3	Dismissed		
			+ Are you bothered by a feeling of heaviness in the genital area during exertion?	24	Component 3	Dismissed		
			+ Are you bothered by a feeling of heaviness in the genital area during sex?	25	Component 3	Dismissed		
Are you bothered by a chafing sensation in the genital area?	-0.2	Dismissed due to CVR < 0						
Are you bothered by pain in the genital area?	0.6	No changes	No changes	26	Component 2	Dismissed		
			+ Are you bothered by pain in the genital area while at rest?	27	Component 2	Dismissed		
			+ Are you bothered by pain in the genital area during exertion?	28	Component 2	Dismissed		
			+ Are you bothered by pain in the genital area during sex?	29	Component 2	Retained	*r* < 0.6 for all items	Retained
Are you bothered by pain in the vaginal opening when something enters the vagina?	0.6	No changes	No changes	30	Component 2	Dismissed		
			+ Have you ever experienced a vaginal orgasm?	31	Not included in PCA due to binary response			
Are you having difficulties experiencing a vaginal orgasm?	1	No changes	No changes	32	Not included in PCA due to missing data (> 50%)			
Are you experiencing genital discomfort that limits your sexual activity?	1	No changes	No changes	33	Component 5	Retained	*r* < 0.6 for all items	Retained
		+ Has your libido/sexual desire decreased due to changes in your genital area after delivery?	*Are you experiencing genital problems that limit your libido (sexual desire)?	34	Component 5	Dismissed		
			+ Do you have penetrative sex?	35	Not included in PCA due to binary response			
			+ Are your genital problems the reason you avoid penetrative sex?	36	Not included in PCA due to binary response			
Are you bothered by pain in the genital area when you have sex?	0.6	No changes	*Are you bothered by pain in the genital area when you have penetrative sex?	37	Component 2	Dismissed		
Are you bothered by pain in the genital area after you have sex?	0.2	No changes	* Are you bothered by pain in the genital area after penetrative sex?	38	Component 9	Dismissed		
Do you have decreased sensation in the genitals during sex?	1	No changes	*Do you have decreased sensation in the genitals during penetrative sex?	39	Component 5	Dismissed		
			+ Do you have increased sensation in the genitals during penetrative sex?	40	Component 7	Dismissed		
Are you experiencing genital discomfort that affects your quality of life?	1	No changes	No changes	41	Component 3	Retained based on clinical relevance	*r* < 0.6 for all items	Retained

*CVR* Content validity ratio, CVR > 0 items retained, *: Rephrased item, + : Added item. PCA = principal component analysis; Items with the highest absolute loadings on each component and items with absolute loadings > 0.55 were retained. If two or more items correlated highly, using a threshold of r > 0.6, selection of these items was once again discussed in a round table session. The final choice was based on interpretable results and clinical benefit

### Setting and study sample

Between 2017 and 2020, women who were referred to the Karolinska University Hospital Pelvic Floor Center with PFD symptoms and diagnosed with a deficient perineum by one of four urogynecologists were invited to the study. Exclusion criteria were non-Swedish speaking women and women planned for combined surgery e.g., prolapse and perineal reconstruction. In total, 177 women were approached, and 170 were included. The control groups were chosen to exclude vaginal birth and pregnancy respectively as confounders. Inclusion criteria were: understand written Swedish, non-pregnant and of fertile age. Thus, primiparous women with one elective caesarean section (CS) due to breach position or non-medical reason between the years 2015–2017 were recruited in 2018, when at least on year had passed since the elective CS. They were identified and contacted through the surgery planning program at the Hospital of Västmanland, Västerås and Karolinska University Hospital, Stockholm, Sweden. 143 women were approached, and 54 women met the inclusion criteria (primipara, non-pregnant and between 25 –45 years old). In addition, non-pregnant nulliparas of fertile age were recruited at the Department of Gynecology at Karolinska University Hospital, Stockholm, Sweden and through social media. 344 women were recruited but six were excluded due to on-going pregnancies. All study participants answered the preliminary 41-item inventory.

### Instrument evaluation

#### Principal component analysis

The preliminary 41 item inventory was administered to 170 eligible patients, 54 primiparous women with elective CS, and 338 nulliparous women. To reduce the number of items of the preliminary 41-item-inventory, principal component extraction using PCA was performed followed by item reduction based on correlation statistics and clinical reasoning. A total of four items (22, 23, 24 36) were excluded before PCA due to limited variance. Kaiser–Meyer–Olkin (KMO), indicating that PCA may be useful with the data, and Bartlett's test of sphericity which tests the hypothesis that the correlation matrix of items is an identity matrix, confirmed that the dataset was appropriate for PCA. The value of KMO was 0.80 and *p*-value for Bartlett's test was < 0.001. All components with an eigenvalue greater than 1 were extracted. Varimax rotations were then performed as they maximize the variance between the components, which simplifies the interpretations of the component solutions. The statistical analyses were performed in R version 4.0.4 (R Development Core Team, 2021).

### Clinical round table

Instead of only selecting items with the highest loadings, a pragmatic selection of items was employed by combining results from PCA with clinical reasoning. Thus, four gynecologists in the research team (GT; SK; ER; HE) discussed the items in a clinical round table setting, applying the following four criteria to select one or two items per component. First, items with the highest absolute loadings on each component were retained. Second, any other items with absolute loadings > 0.55 were also retained [23]. Third, the four gynecologists were allowed to add one more item that was not selected based on the first two criteria but added clinical value. Fourth, high correlations between items, based on the Pearson correlation coefficient, were avoided. If two or more items correlated highly, using a threshold of *r* > 0.6, selection of these items was once again discussed in a round table session.

### Construct validity; comparison to control groups

Using a non-parametric procedure, we employed Mann–Whitney U-tests to compare each control group with the Patient group. Median values and inter-quartile range (IQR) were used to describe the distribution of total scores. Bootstrap percentile 95% confidence intervals (95% CI) were constructed based on drawing and replacing 2,000 bootstrap replicates, to compare differences in total score between the Patient group and each control group (Elective CS and Nullipara).

## Cut-off values

Receiver operating characteristic (ROC) curves were conducted to investigate the discriminative ability of the inventory as well as to determine the optimal cut-off points for the final inventory to distinguish patients from the Elective CS and Nullipara groups.

## Results

### Characteristics of the study samples

Mean patient age was 37.4 years (SD 6.6) with mean BMI 23.8 kg/m^2^ (SD 4.8). Mean parity, defined as all previous pregnancies that resulted in a live birth or a stillbirth of > 22 weeks gestation, was 2.2 (SD 0.8) children and the group had a mean of two vaginal births per patient (SD 0.9). The Nullipara group (*n* = 338) were significantly younger (*p* < 0.05) than the Patient group, mean age 31.4 years (SD 7.5) with higher mean BMI 24.9 (SD 6.1), but not significant (*p* = 0.06). Women with elective CS were also significantly younger than the Patient group (*p* < 0.05) with a mean age of 34.9 years (SD 4.4) and a mean parity of 1. BMI for Elective CS was not obtained.

### Instrument evaluation

#### Psychometric analysis

In total, 170 patients completed the preliminary 41-item-inventory. Based on the PCA, a ten-component solution was chosen, explaining a cumulative variance of 70% (Supplementary file). The PCA results were then discussed in a clinical round table and 11 items were retained (1–2 items per component), and one clinically relevant item from the preliminary inventory was added (Table 1). Based on correlation statistics and round table discussions, one item was then excluded (Table 2), hence, two components (8 and 10) were not represented (6.2% of the total variance).

**Table 2:** The 11-item Karolinska Symptoms After Perineal Tear Inventory with a total score of 33 points

Number	Item
1	Do you feel that your vagina is too wide/loose?^a^
2	Do you have a feeling of looseness deep inside the vagina?^a^
3	Are you bothered by air entering the vagina?^b^
4	Are you bothered by sounds caused by air escaping from the vagina (vaginal flatulence)?^b^
5	Are you bothered by a feeling of heaviness in the genital area?^b^
6	Are you bothered by leakage of loose stool?^b^
7	Do you need to use your fingers to apply pressure from inside the vagina or around the anus to defecate? ^b^
8	Do you have to sit or stand in a particular position to be able to defecate?^b^
9	Are you bothered by pain in the genital area during sex?^b^
10	Are you experiencing genital discomfort that limits your sexual activity?^a^
11	Are you experiencing genital discomfort that affects your quality of life?

^a^ Response option (4-point scale): Strongly disagree = 0, Disagree = 1; Agree = 2, and Strongly agree = 3

^b^ Response option (4-point scale): Never = 0, Sometimes = 1, Often = 2, and Always = 3

### The final inventory

The final version of the inventory “Karolinska Symptoms After Perineal Tear Inventory" (KAPTAIN) consisted of 11 items with 1–2 items per symptom domain, with a total score range of 0–33 (Table 2). A higher score indicates more symptoms and increased symptom bother. The mean KAPTAIN score of the Patient group was 19.0 (SD 4.4), with mean score per item 1.7 (SD 0.4).

#### Construct validity: Comparison to control groups

**Fig. 1:**
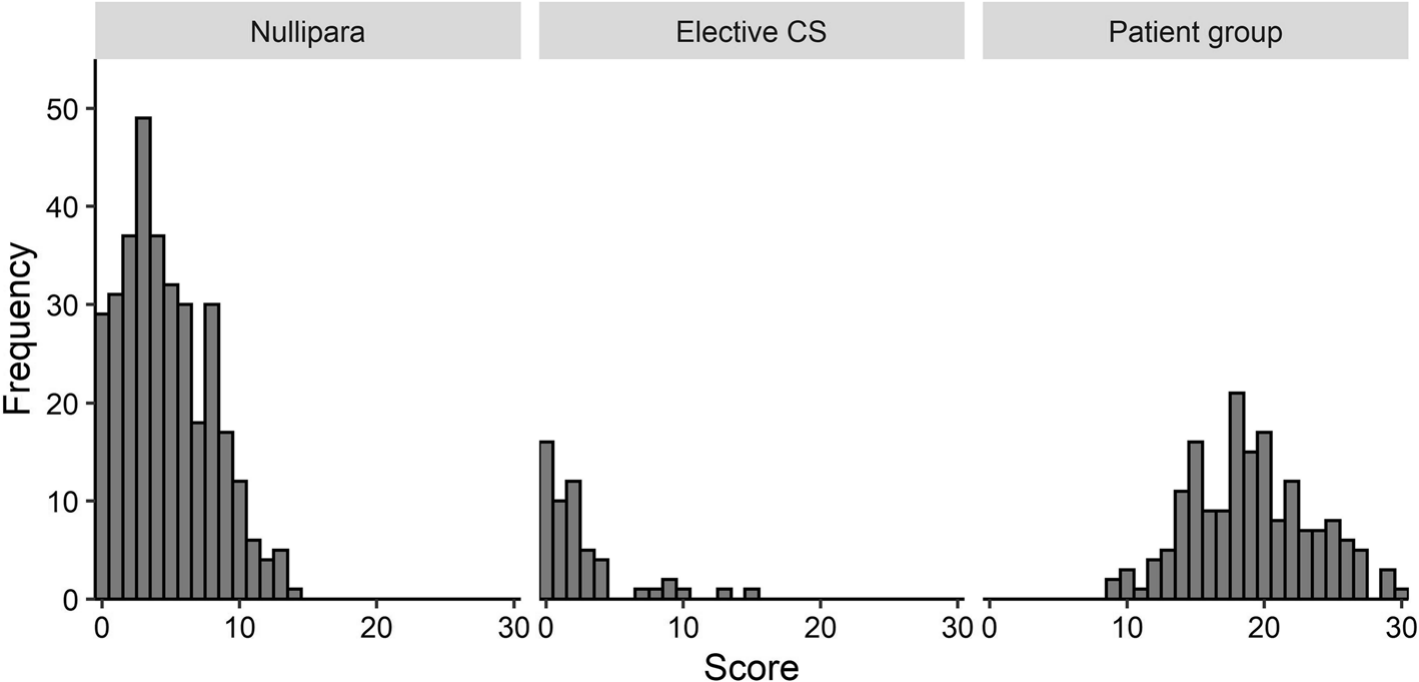
The distribution of frequencies and total scores of the final 11-items inventory by groups

Mann–Whitney U-tests showed that the Patient group (median 19, IQR 16–22) had a significantly (*p* < 0.001) higher score on the final 11-item inventory, compared to the Elective CS (median 2, IQR 0–3) and Nullipara group (median 4, IQR 2–7). Based on 2,000 bootstrap samples, the 95% CI for score differences between the Patient group, compared to Elective CS, was 15.4–17.6, and 13.6–15.1 compared to Nullipara group (Table 3). The distribution of the total score of the final 11-item inventory for the three groups is presented in Fig. 1.

**Table 3:** Total score on “Karolinska Symptoms After Perineal Tear Inventory” (KAPTAIN) and mean differences for Patient group, Nullipara and Elective CS based on bootstrap sampling. *P*-value calculated using Mann–Whitney U-test

	Mean score (SD)	Median score (IQR)	Comparisons	Mean differences (95% CI)	*P*-value
Patient group	19.0 (4.4)	19 (16–22)	Patient group vs. Nullipara	14.4 (13.6–15.1)	< 0.05
Nulliparae	4.7 (3.2)	4 (2–7)	Nullipara vs. Elective CS	2.1 (1.2–3.0)	< 0.05
Elective CS^a^	2.5 (3.3)	2 (0–3)	Patient group vs. Elective CS	16.5 (15.4–17.6)	< 0.05

*CI* Confidence intervals, *IQR* Inter-quartile range, *SD* Standard deviation

^a^Skewness 2.1

### Cut-off values

**Fig. 2:**
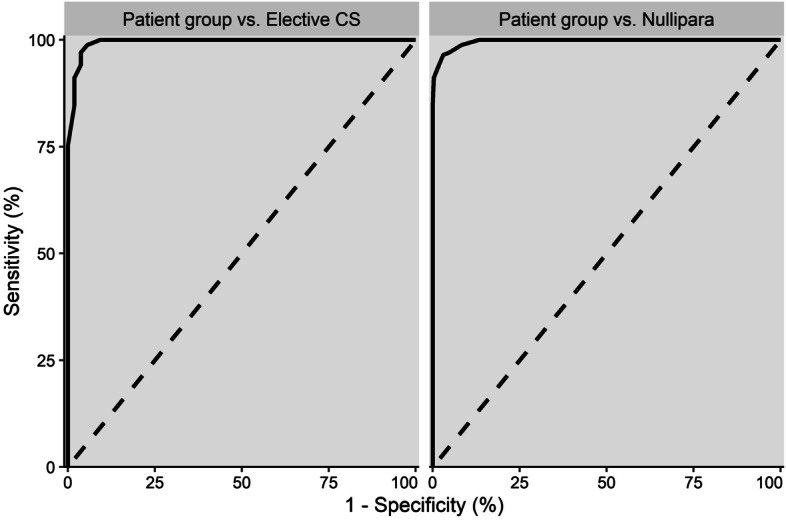
ROC curve demonstrating the relationship between sensitivity and specificity values for classifying the Patient group compared to Elective CS and Nullipara, when varying the cut-off score. Dashed line as reference

The ROC curves yielded an optimal cut-off value of  ≥ 8 as this could distinguish the target Patient group from the Elective CS with a sensitivity of 100% and, specificity of 91%. Applying the same cut-off value could separate the patients from nulliparous women with a sensitivity and specificity of 100% and 87%, respectively (Fig. 2).

## Discussion

The overarching aim of this study was to construct and psychometrically evaluate a Swedish inventory for women with symptoms of deficient perineum after vaginal birth. We have described the development process of a conceptual framework based on a qualitative approach and psychometric analysis, including literature review, expert panel, and PCA in combination with clinical round table discussions to determine relevant items [24–26].

The validation process shows that the inventory is psychometrically stable and can distinguish symptomatic women with deficient perineum from women in the control groups with a sensitivity of 100% and specificity of 87–91%. Furthermore, response distributions for each item were analyzed with no significant floor or ceiling effects, indicating that KAPTAIN covers a wide range of PFD symptoms.

Given the relatively high prevalence of perineal tears [27, 28] and the scarcity of inventories concerning PFD symptoms more than one year after childbirth [10], the KAPTAIN inventory is a promising new instrument and a valuable addition to the clinical assessment. Moreover, there are several national quality registers in Sweden with the aim to improve and follow-up health care outcome. In addition to the Swedish National Quality Registry of Obstetric Tears, women are, offered a registry-based follow-up through the National Medical Birth Registry irrespective of obstetric tear, with the aim of identifying women that need further gynecological assessment, nevertheless, all women with symptoms of deficient perineum are not identified today as a validated screening inventory is lacking. In a review by Zuchelo et al., nine validated instruments for assessing PFD were identified [10], but none of them assesses PFD after vaginal birth in a complete and integrated way, as they are designed for the general population or for patients with incontinence. As most pelvic floor symptoms can be traced back to the progress of vaginal birth, Luthander et al. [29] declare the need for validated instruments to feedback the obstetric management. Furthermore, Barber et al. highlight that the evaluation of persistent pelvic floor symptoms after vaginal birth requires validated questionnaires [30] and Voorham-van der Zalm et al. [31] state that inventories are useful tools in research. Thus, the KAPTAIN might prove to be a valuable tool for both management and research while also enabling a comprehensive strategy for health care providers to detect women in need of counselling or further intervention after vaginal birth.

A strength of the study is the framework used to ensure both content validity and psychometric evaluation [15]. In addition, the repeated advice of an expert panel to identify potential items was used to ensure the clinical relevance when selecting the individual items, as well as the decision of a cut-off score [26]. Nonetheless, there are some limitations that need to be addressed. First, data from patients were collected within a tertiary setting that limits generalizability. Second, by using control groups of women to exclude pregnancy as a confounder, we cannot draw the conclusion that this inventory only identifies women with symptoms of deficient perineum. However, we can state that it distinguishes women who have subjective pelvic floor symptoms after vaginal birth from women who have been pregnant and given birth by caesarean section and from women who have not been pregnant or given birth. Third, BMI are missing for the Elective CS group as unfortunately no demographic characteristics were obtained except to control for inclusion criteria. However, since the group scores significantly lower than the Patient group BMI might not be a confounder affecting the scoring. Fourth, we only used gynecologist as experts for the round table discussions as women who present with persistent symptoms are primarily referred to urogynecologists for evaluation in Sweden. Nevertheless, it might have been of value to include other health care professionals or patient representatives in the selection of items. In addition, KAPTAIN is only validated in women who understand written Swedish.

KAPTAIN was developed to discern women with PFD symptoms due to deficient perineum after obstetric perineal trauma. Despite this, this inventory must be related to gynecological and obstetric history as well as results of a gynecological exam to offer correct diagnosis and treatment. The procedure of using inventories prior to a clinical examination is well used and successful in managing many other areas of pelvic floor medicine [32, 33]. It should be noted that this study does not compare symptoms after different ways of vaginal delivery, as the Patient group is not representative of all women after vaginal childbirth. We have only included a subgroup of women with confirmed deficient perineum, thus all our patients had symptoms and therefore our sample does not mirror all women with perineal trauma after vaginal birth.

We hope that KAPTAIN will be useful to clinicians and researchers alike. However, whether cross-cultural differences exist, whether different populations have different mean scores, if specific symptoms will be more prognostic, and whether separate norms will affect the results are important questions that need to be addressed in future research. Moreover, we plan to further evaluate the inventory in a large cohort of women diagnosed with perineal tears to verify that the inventory can distinguish women with symptoms of deficient perineum who might benefit from perineal reconstruction or other intervention. In addition, reliability will be evaluated by performing test–retest to ensure that the inventory can replicate the result more than once in the same situation and population [34]. Furthermore, to use the inventory to evaluate interventions such as reconstructive surgery, it needs to be assessed in respect to responsiveness, or sensitivity to change. This includes the ability of an instrument to detect a small but clinically important change [35]. Finally, the International Consultation on Incontinence encourages researchers to translate and validate instruments in different languages to allow for cross-cultural research [36]. This would give us the possibility to design larger studies with international participation.

## Conclusions

The KAPTAIN is a psychometrically stable inventory that measures long-term subjective symptoms in women with a deficient perineum after vaginal childbirth. KAPTAIN can be used to screen for women at risk of PFD after vaginal birth and, in a future perspective, may be used to identify patients who might benefit from intervention.

## Supplementary Information

**Additional file 1.**

## Data Availability

All authors had full access to the data and materials. The datasets generated and/or analysed during the current study are not publicly available due to institutional restrictions but are available from the corresponding author on reasonable request.

## Abbreviations

KAPTAIN: The Karolinska symptoms after perineal tear inventory
PFD: Pelvic floor dysfunction
CVR: Content validity ratio
CS: Caesarean section
PCA: Principal component analysis
ROC: Receiver operating characteristic
