# Supplementary material for: Development and initial validation of a Swedish inventory to screen for symptoms of deficient perineum in women after vaginal childbirth: ‘Karolinska Symptoms After Perineal Tear Inventory’
Source: BMC Pregnancy Childbirth. 2022 Aug 13;22:638. doi: 10.1186/s12884-022-04964-w (PMC9375344; doi:10.1186/s12884-022-04964-w)
Supplement: Supplementary file 1 — Additional file 1. [file 12884_2022_4964_MOESM1_ESM.docx]

**Supplementary file**

**Table 1**. Component loadings from principal component analysis based on varimax rotation for a ten-component solution. Items with loadings >0.55 are marked in bold.

| **Items** | **Component** | | | | | | | | | |
| --- | --- | --- | --- | --- | --- | --- | --- | --- | --- | --- |
|  | 1 | 2 | 3 | 4 | 5 | 6 | 7 | 8 | 9 | 10 |
| Do you feel that your vagina is too wide/loose? | 0.203 | -0.091 | 0.255 | -0.079 | -0.043 | -0.257 | **0.602** | 0.171 | -0.026 | 0.032 |
| Do you have a feeling of looseness deep inside the vagina? | 0.192 | 0.082 | 0.257 | -0.082 | 0.236 | 0.194 | **0.646** | 0.075 | 0.023 | -0.087 |
| Are you experiencing discomfort because you feel your vaginal opening is exposed or unprotected? | 0.453 | 0.011 | 0.312 | 0.002 | 0.061 | -0.085 | 0.302 | 0.141 | 0.000 | 0.427 |
| Are you experiencing that your genital area is cold because your vaginal opening is exposed or unprotected? | 0.360 | 0.085 | 0.234 | -0.016 | -0.071 | -0.019 | 0.098 | -0.156 | 0.034 | **0.720** |
| Are you bothered by air or a sensation of draft in the genital area? | **0.723** | 0.069 | 0.022 | -0.040 | -0.048 | -0.046 | 0.144 | -0.005 | -0.030 | 0.299 |
| Are you bothered by air entering the vagina? | **0.840** | 0.087 | 0.067 | 0.076 | 0.037 | -0.048 | 0.095 | 0.152 | -0.071 | 0.017 |
| Are you bothered by air entering the vagina when you are at rest? | **0.703** | 0.154 | 0.062 | -0.027 | 0.143 | 0.203 | 0.129 | -0.145 | 0.056 | -0.082 |
| Are you bothered by air entering the vagina during exertion? | **0.746** | 0.147 | 0.267 | 0.109 | 0.165 | -0.005 | 0.051 | -0.013 | -0.050 | 0.020 |
| Are you bothered by air entering the vagina when you have sex? | **0.600** | 0.145 | 0.101 | 0.143 | 0.107 | 0.042 | 0.182 | **0.591** | 0.087 | -0.079 |
| Are you bothered by sounds caused by air escaping from the vagina (vaginal flatulence)? | **0.774** | 0.019 | -0.031 | -0.031 | 0.026 | 0.124 | 0.112 | 0.207 | -0.017 | 0.018 |
| Are you bothered by vaginal flatulence while at rest? | **0.698** | 0.010 | 0.001 | -0.053 | 0.117 | 0.313 | 0.023 | -0.170 | 0.111 | 0.179 |
| Are you bothered by vaginal flatulence during exertion? | **0.802** | 0.029 | 0.164 | -0.019 | 0.112 | 0.021 | -0.078 | 0.043 | -0.040 | 0.100 |
| Are you bothered by vaginal flatulence during sex? | **0.575** | 0.082 | 0.115 | 0.197 | 0.063 | 0.056 | 0.111 | **0.609** | 0.038 | -0.166 |
| Do you experience difficulties defecating? | -0.001 | 0.108 | 0.099 | **0.735** | 0.033 | 0.272 | -0.062 | 0.144 | 0.173 | 0.071 |
| Do you need to use your fingers to apply pressure from inside the vagina or around the anus to defecate? | 0.002 | -0.072 | 0.117 | 0.217 | 0.114 | -0.049 | -0.037 | 0.094 | **0.814** | 0.089 |
| Do you need to use your fingers to apply pressure from inside the vagina or around the anus to release anal flatulence? | -0.056 | 0.028 | 0.005 | 0.429 | 0.244 | 0.131 | -0.370 | 0.079 | 0.341 | 0.058 |
| Do you have to sit or stand in a particular position to be able to defecate? | 0.076 | 0.171 | 0.103 | **0.880** | 0.031 | 0.008 | -0.077 | 0.089 | 0.145 | 0.007 |
| Does it make it easier to defecate if you sit or stand in a particular position? | -0.011 | 0.053 | 0.052 | **0.834** | -0.049 | -0.078 | 0.063 | -0.097 | -0.051 | -0.073 |
| Are you bothered by flatus incontinence? | 0.124 | 0.041 | 0.037 | 0.230 | -0.239 | **0.717** | 0.097 | 0.060 | -0.160 | -0.096 |
| Are you bothered by leakage of loose stool? | -0.002 | -0.001 | 0.105 | 0.025 | 0.050 | **0.807** | -0.041 | 0.049 | -0.053 | -0.002 |
| Are you bothered by leakage of solid stool? | 0.201 | 0.147 | 0.060 | -0.043 | 0.106 | **0.638** | -0.060 | -0.042 | 0.106 | 0.051 |
| Are you bothered by a feeling of heaviness in the genital area? | 0.112 | 0.077 | **0.873** | 0.120 | 0.176 | 0.012 | 0.090 | 0.032 | 0.140 | 0.035 |
| Are you bothered by a feeling of heaviness in the genital area while at rest? | 0.091 | 0.309 | **0.677** | 0.028 | 0.120 | 0.260 | 0.043 | -0.112 | 0.110 | 0.146 |
| Are you bothered by a feeling of heaviness in the genital area during exertion? | 0.163 | 0.114 | **0.866** | 0.097 | 0.113 | -0.042 | 0.103 | 0.015 | 0.032 | 0.054 |
| Are you bothered by a feeling of heaviness in the genital area during sex? | 0.112 | 0.281 | **0.670** | 0.056 | 0.238 | 0.259 | 0.045 | 0.201 | 0.107 | 0.128 |
| Are you bothered by pain in the genital area? | 0.204 | **0.765** | 0.231 | 0.118 | 0.070 | 0.018 | 0.053 | -0.242 | 0.118 | -0.092 |
| Are you bothered by pain in the genital area while at rest? | 0.123 | **0.715** | 0.231 | 0.250 | 0.107 | 0.015 | 0.145 | -0.146 | 0.117 | -0.086 |
| Are you bothered by pain in the genital area during exertion? | 0.175 | **0.681** | 0.277 | 0.192 | 0.093 | -0.012 | 0.044 | -0.275 | 0.191 | 0.044 |
| Are you bothered by pain in the genital area during sex? | 0.072 | **0.801** | 0.016 | 0.101 | 0.061 | 0.161 | -0.130 | 0.329 | 0.002 | 0.140 |
| Are you bothered by pain at the vaginal opening when something enters the vagina? | 0.000 | **0.823** | -0.006 | -0.130 | 0.092 | 0.011 | -0.121 | 0.105 | -0.070 | -0.007 |
| Are you experiencing genital discomfort that limits your sexual activity? | 0.175 | 0.277 | 0.178 | -0.013 | **0.781** | 0.030 | 0.131 | 0.007 | -0.036 | -0.001 |
| Are you experiencing genital problems that limit your libido (sexual desire)? | 0.141 | 0.153 | 0.215 | -0.026 | **0.809** | -0.081 | 0.007 | 0.069 | 0.051 | -0.014 |
| Are you bothered by pain in the genital area when you have penetrative sex? | 0.066 | **0.750** | 0.106 | 0.097 | 0.131 | 0.119 | -0.257 | 0.294 | -0.028 | 0.104 |
| Are you bothered by pain in the genital area after penetrative sex? | -0.048 | 0.281 | 0.166 | 0.032 | -0.203 | -0.038 | 0.027 | -0.075 | **0.714** | -0.089 |
| Do you have decreased sensation in the genitals during penetrative sex? | 0.117 | -0.039 | 0.143 | 0.131 | 0.491 | 0.233 | 0.369 | 0.243 | -0.031 | 0.381 |
| Do you have increased sensation in the genitals during penetrative sex? | -0.098 | 0.205 | 0.087 | -0.027 | -0.117 | -0.003 | **-0.724** | 0.095 | 0.009 | -0.206 |
| Are you experiencing genital discomfort that affects your quality of life? | 0.201 | 0.042 | 0.284 | 0.208 | 0.453 | 0.027 | 0.125 | -0.283 | -0.116 | -0.216 |
|  |  |  |  |  |  |  |  |  |  |  |
| Cumulative variance (%) | 23.7 | 35.1 | 42.2 | 48.7 | 53.9 | 58.3 | 61.8 | 65.3 | 68.1 | 70.8 |
